# Does poor oral health impact on young children's development? A rapid review

**DOI:** 10.1038/s41415-024-7738-4

**Published:** 2024-08-23

**Authors:** Samantha Watt, Tom A. Dyer, Zoe Marshman, Kate Jones

**Affiliations:** 143185680435168940182 Academic Clinical Fellow and Specialty Registrar in Dental Public Health, School of Clinical Dentistry, University of Sheffield, United Kingdom; 941052077760540001698 Senior Clinical Teacher in Dental Public Health, School of Clinical Dentistry, University of Sheffield, United Kingdom; 986172231275938144256 Professor of Dental Public Health, School of Clinical Dentistry, University of Sheffield, United Kingdom; 736783457909472812691 Consultant in Dental Public Health, Office for Health Improvement and Disparities, London, United Kingdom

## Abstract

**Background** The extent to which poor oral health in young children impacts developmental milestones is unclear.

**Aim** To review the evidence of association between poor oral health and development of young children (0-5 years).

**Method** A rapid review was conducted. Medline was searched for articles in English published from 2000 onwards. The outcomes considered were the impact of poor oral health on speech and language development, oral health-related quality of life (OHRQoL), school performance and attendance.

**Results** In total, 15 studies were included: four systematic reviews, a critical review and 10 primary studies. All data were from observational or cross-sectional studies. Premature extraction of primary maxillary incisors was associated with change in speech sounds and articulation. Two primary studies found associations between early childhood caries and physical and psychomotor deficiencies, while acknowledging a web of causation with other factors, including socioeconomic status. Children with dental caries reported lower OHRQoL. Studies found caries experience was associated with preschool absenteeism.

**Conclusion** Poor oral health in young children appears to be associated with speech and language development, OHRQoL, and school attendance and performance. However, there are significant limitations in the existing evidence, with further high-quality, observational, longitudinal research required to fully understand the impact of poor oral health on the development of young children. Strategies to improve oral health by health and education professionals are needed to ensure young children reach their potential.

**Supplementary Information:**

Zusatzmaterial online: Zu diesem Beitrag sind unter 10.1038/s41415-024-7738-4 für autorisierte Leser zusätzliche Dateien abrufbar.

## Key points

- This rapid review describes current evidence on the impact of poor oral health on young children's speech and language development, oral health-related quality of life, and school attendance and performance.
- There is some evidence that poor oral health can affect young children's developmental potential. However, high-quality, observational, longitudinal research is required to improve the strength of the evidence.
- Strategies to improve the oral health of young children are required to ensure they reach their full developmental potential.

**Supplementary Information:**

Zusatzmaterial online: Zu diesem Beitrag sind unter 10.1038/s41415-024-7738-4 für autorisierte Leser zusätzliche Dateien abrufbar.

## Introduction

The World Health Organisation identified socially marginalised groups as bearing the highest burden of oral disease, with children being most vulnerable.^1^ Poor oral health in young children is most commonly due to early childhood caries (ECC). ECC is defined as ‘the presence of one or more decayed (non-cavitated or cavitated lesions), missing or filled (due to caries) surfaces in any primary tooth of a child under six years of age'.^2^ Although prevalence varies within and between nations, ECC affects nearly half of preschool children globally.^3^ As well as affecting comfort, function and ultimately, quality of life, the debilitating effects of poor oral health may also extend to a child's physical, cognitive and psychosocial development.^4^ However, the extent to which poor oral health in young children impacts developmental milestones, such as speech and language, is unclear.

Moreover, children with speech and language difficulties at age five years are more likely to have difficulty reading, experience negative impacts on adolescent mental health and be unemployed as adults.^5^^,^^6^ If poor oral health also affects preschool and school attendance, any effects are likely to be exacerbated.^4^^,^^6^ Learning and development experiences in the early years are seen as crucial in providing the foundation for success in school, adolescence and life.^6^ Therefore, if there is evidence that poor oral health is associated with developmental milestones, it would be useful for policymakers and professionals working in health, education and childcare.

Indeed, the oral health of children has been recognised recently as a priority for improvement in England, both by health and education government departments. NHS England has developed the Core20PLUS5 approach to support the reduction of inequalities in children and young people, with oral health identified as a key clinical area of health inequality.^8^ Oral health is also included in the Early Years Foundation Stage framework, with a justification for its inclusion to support speech and language development.^9^ An understanding of the impact of poor oral health on young children's development will help to inform further relevant national and local policies.

The aim of this study was to conduct a rapid review of the evidence of association between poor oral health in young children and speech and language development, including oral health-related quality of life (OHRQoL) and school attendance and performance, to inform national and local policy on child oral health improvement.

## Methods

Rapid reviews synthesise knowledge to produce information for policymakers in short timeframes.^10^^,^^11^^,^^12^ Although methods vary, they simplify or omit stages of conventional systematic reviews by limiting the number and scope of questions, searching fewer databases, reducing hand-searching and simplifying evidence synthesis.^13^ A protocol was not published prior to undertaking the review in line with similar published rapid reviews.

The population of interest was young children, from birth to five years of age, across all socioeconomic groups. This age range was chosen as the target population as this is included in the statutory framework for the early years foundation stage in the UK.^5^ The exposure of interest was children with poor oral health, defined as experience of dental caries or premature tooth loss. The primary outcome was the impact of poor oral health on speech and language development. Secondary outcomes were OHRQoL and school performance and attendance.

Only English-language, full-text papers published from 2000-2023 were included. This period was selected to ensure findings were relevant to current populations. There was no limitation on research setting as it was assumed the impacts of poor oral health on primary and secondary outcomes would be similar globally. Synthesised evidence (secondary research) was prioritised for inclusion; however, when unavailable, sources of primary research were included. Studies were excluded if related to cranio-facial conditions or syndromes, if participants had co-morbidities and if studies evaluated the management or prevention of caries. Studies were also excluded if related to the sequelae of trauma but included if related to the sequelae of caries and trauma.

A simplified search limited to Medline via Ovid was undertaken on 31 March 2023. The search strategy used a combination of free-text search terms, applied Boolean operators (AND and OR), and controlled vocabulary subject headings to obtain comprehensive record retrieval (see online Supplementary Information).

Having conducted the search, identified records were exported into Rayyan in RIS format, de-duplicated and screened. A single author (SW) screened all records to select studies for inclusion. Any queries in the suitability of a study for inclusion were discussed with a second author (ZM). A single author (SW) extracted data, using a pre-defined data extraction form, including study design; location; characteristics of setting and population (age, oral disease status, ethnicity, socioeconomic status); follow-up duration; and the assessed/reported outcomes relevant to the review's scope (ie data on speech and language, OHRQoL and school attendance and performance).

A narrative synthesis was undertaken. Relevant quantitative data were reported describing effect size, direction and any variation. Quantitative synthesis, sensitivity analyses and formal assessment of bias were not undertaken due to the heterogeneity of methods and outcomes of included studies; however, methodological strengths and weaknesses of studies were identified and discussed.^14^

## Results

**Fig. 1:**
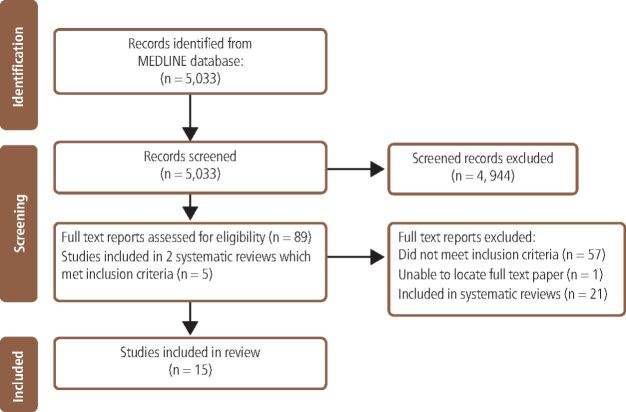
PRISMA diagram of literature search process

The search identified 5,033 records. All titles and abstracts were screened and 4,944 were deemed ineligible for inclusion. Following full-text screening of 89 records, ten were included in the study (Fig. 1). A further five primary studies from two systematic reviews that met the inclusion criteria were also included. All synthesised and primary data were from observational and cross-sectional studies. See Table 1 for a summary of the included studies.

**Table 1:** Summary of included studies

Study	Year	Country	Study type	Indicator of oral health (measure)	Primary or secondary outcome (measure)
Nadelman *et al*.^15^	2020	Brazil	Systematic review and meta-analysis	Premature loss or extraction of primary maxillary incisors	Primary: consequences to speech and dental arch perimeter
Adewumi *et al*.^16^	2012	USA	Cohort	Premature loss or extraction of primary maxillary incisors	Primary: parental responses to telephone interview questions about speech changes following extraction of primary maxillary incisors and professional speech evaluation
Turgut *et al*.^17^	2012	Turkey	Case-control	Premature loss of at least one primary maxillary incisor	Primary: speech articulation assessed by a speech therapist
Nadelman *et al*.^18^	2015	Brazil	Critical review	Premature loss of primary anterior teeth	Primary: speech impairment, development of non-nutritive habits, psychosocial consequences
Liang *et al*.^19^	2022	Taiwan	Cohort	Caries (dmft using WHO criteria)	Primary: psychomotor development (psychomotor development and the CCDI scales)
Liang *et al*.^20^	2019	Taiwan	Cross sectional	Caries (dmft using WHO criteria)	Primary: psychomotor development (psychomotor development and the CCDI scales)
Zaror *et al*.^21^	2022	Chile	Systematic review and meta-analysis	Caries (dmft using WHO criteria and ICDAS II)	Secondary: oral health-related quality of life (ECOHIS, SOHO5, PPQ, OIDP, PedsQL and OHECQOL)
Nora *et al*.^22^	2018	Brazil	Systematic review and meta-analysis	Caries (dmft using WHO criteria and ICDAS II)	Secondary: oral health-related quality of life (ECOHIS, SOHO5, PPQ, POQL, PedsQL and ITQOL)
Rebelo *et al*.^23^	2019	Brazil	Systematic review and meta-analysis	Caries (dmft using WHO criteria and ICDAS II) Other measures: gingivitis, dental trauma and malocclusion Parent's perception of child's oral health, self-perceived oral health and toothache	Secondary: school performance and attendance
Neves *et al*.^24^	2016	Brazil	Cross-sectional	Caries (ICDAS II), traumatic dental injury and malocclusion	Secondary: oral health-related quality of life (ECOHIS)
Gradella *et al*.^25^	2011	Brazil	Cross-sectional	Caries (dmft) and its consequences (PUFA index: P = visible pulp involvement; U = ulceration of oral mucosa owing to root fragments; F = fistula; A = abscess)	Secondary: Brazilian version of the PPQ for preschool children
Anandakrishna *et al*.^26^	2012	India	Cross-sectional	Caries (dmft using WHO criteria)	Secondary: academic performance based on marks obtained. Children categorised as excellent (>95% marks), average (50-95% marks), below average (<50%)
Janus *et al*.^27^	2019	Canada	Cross-sectional	Teacher-reported unaddressed dental needs	Secondary: academic development (The Early Development Instrument - 103-item teacher-completed questionnaire measuring child's ability to meet age-appropriate developmental expectations prior to entering Grade 1)
Nasuuna *et al*.^28^	2016	Australia	Cross-sectional	Parent-reported dental problems	Secondary: school readiness (NAPLAN - measure of academic performance where students assessed on numeracy, reading, writing, grammar and spelling using standard tests at Years 3, 5, 7 and 9 of school)
Muirhead *et al*.^29^	2004	England	Ecological study	Caries (dmft using WHO criteria)	Secondary: school performance - school performance results in English, mathematics and LARR (literacy)
Key: WHO = World Health Organization ICDAS = International Caries Detection and Assessment System ECOHIS = Early Childhood Oral Health Impact Scale SOHO5 = Scale of Oral Health Outcomes for Five-Year-Old Children PPQ = Parental Perceptions Questionnaire POQL = Paediatric Oral Health-Related Quality of Life PedsQL = Paediatric Quality of Life Inventory ITQOL = Infant/Toddler Quality of Life Questionnaire					

### Speech and language development

A systematic review of observational studies aimed to evaluate the consequences of premature loss of primary anterior teeth in children's speech and arch integrity.^15^ Two of the included studies met the inclusion criteria of this review. A US case-control study reported that, following premature loss of maxillary primary teeth, parents perceived that their child's speech sounded different, they experienced difficulty articulating certain sounds (eg ‘s' and ‘z') and they had difficulty eating and chewing. In addition, there was agreement between parental perceptions and actual disarticulations detected by professional assessment.^16^ A case-control study conducted in Turkey investigated the effects of primary anterior tooth loss and dentures on the speech of children with ECC. Although dentures initially affected articulation of certain sounds, participants compensated and articulated speech sounded correct at a follow-up appointment.^16^ Both studies had very small samples and were assessed as high risk of bias.^15^ A critical review by the same authors emphasised premature loss of primary teeth may cause speech distortion.^18^ However they note the scarcity of recent data and the methodological limitations of published studies.

Two primary studies supported the association between poor oral health and speech and language development delays in preschool children. A cross-sectional survey and cohort study conducted by the same authors in Taiwan evaluated potential associations between higher levels of ECC and physical and/or psychomotor deficiency in children aged 3-6 years.^19^^,^^20^ Data on caries experience (decayed, missing and filled primary teeth [dmft]), diet, body mass index, psychomotor development and the Chinese Child Development Inventory (CCDI) scale were collated. The CCDI is a modification of the Minnesota Child Development Inventory, comprising 320 items over seven developmental areas (gross motor, fine motor, expressive language, comprehension concept, situation comprehension, self-help, personal-social) and one summary scale. The cross-sectional study reported a positive correlation between severe ECC (dmft >3-8) and psychomotor deficiency (expressive language and comprehension concept scales). Regression analyses using CCDI developmental areas as dependent variables identified a statistically significant relationship between expressive language (undefined) and dmft scores (≤2 vs ≥3).^20^ In the cohort study, the authors suggest a causal relationship between ECC and psychomotor deficiency in preschoolers. Having controlled for diet and socioeconomic status, higher ECC (dmft scores: <4 vs 6-10) was associated with psychomotor deficiency (in the development areas of expressive language, comprehension concept, gross motor and self-help). However, the authors noted a web of causation involving socioeconomic status and diet which required further investigation.^19^

### Oral health-related quality of life

Two systematic reviews with meta-analyses of observational studies evaluated the impact of caries on OHRQoL in preschool children.^21^^,^^22^ Both included studies that clinically assessed caries, assessed OHRQoL with a validated instrument and compared the OHRQoL of children with and without caries. Studies that included children with systemic diseases were excluded. Included studies in both reviews were cross-sectional, cohort or case-control design and most were conducted in Brazil.

Zaror and colleagues aimed to assess the impact of ECC on OHRQoL.^21^ They included preschool children under six years but excluded studies that: included other ages and did not stratify results by age; assessed the psychometric properties of an OHRQoL questionnaire; were case reports or series with fewer than ten participants; and studies that reported secondary data.^21^ In total, 35 studies were included in the review: 15 were assessed as methodologically weak, 18 moderate and two were strong. Of the 35 included studies, 24 were included in the meta-analysis, all of which found that ECC negatively impacted the OHRQoL of preschool children. The authors pooled data from studies providing dichotomous results (impact vs no impact). Ten studies showed that children with ECC were more likely to report a negative impact on OHRQoL than those without caries (OR: 3.01; 95% CI: 2.43-3.74; I^2^ = 79%; very low-quality of evidence). Pooled data that had been adjusted for confounders from six studies confirmed children with ECC were more likely to report negative OHRQoL impacts (OR: 1.99; 95% CI: 1.51-2.62; I^2^ = 85%; very low-quality of evidence). A total of 14 studies reported OHRQoL scores, which allowed the standard mean difference (MD) between the ECC group and the control group (those without caries) to be calculated: 0.81; 95% CI: 0.61-1.00; I^2^ = 92% (very low-quality of evidence). In addition, all domains of the Early Childhood Oral Health Impact Scale were impacted in patients with ECC, with the social and psychological domains most affected, although heterogeneity was reported as high. Severe ECC (dmft index >5) was also found to increase the negative impact of OHRQoL in preschool children compared with those with non-severe ECC.^21^

A systematic review and meta-analysis of observational studies pooled data from 12 of 29 included studies.^22^ It aimed to assess if caries negatively impacts the OHRQoL of preschool children (defined as those up to five-year-olds) and excluded studies that did not report sample size calculations. In comparing the mean OHRQoL scores of those without and those with caries, those without had a lower mean score, thus there was a negative mean difference. All 29 included studies found caries negatively impacted OHRQoL. A higher impact was reported for those with dmft ≥1 compared with those without caries (MD: −3.57; 95% CI -5.16 to -1.98; I = 96%). Consistent with the findings of Zaror and colleagues (2022), severity of caries experience correlated negatively with OHRQoL impacts. Children with a dmft ≥6 showed a greater impact on OHRQoL (MD: -9.19; 95% CI -13.00 to -5.38; I^2^ = 95%). The evidence presented in the review was assessed as being at low certainty due to the observational nature of the studies and substantial methodological heterogeneity.^22^

### School attendance and performance

One systematic review evaluated the association between oral health and preschool attendance and performance; three of the included studies related to children aged 2-5 years.^23^^,^^24^^,^^25^^,^^26^ Two of these were cross-sectional studies undertaken in Brazil. The first found cavitated caries was the most common oral health problem among participants and was associated with preschool absenteeism after logistical regression (OR: 2.872; 95% CI: 1.266-6.514; p = 0.012).^24^ The second found caries experience was positively associated with absence from school (OR: 4.38, 95% CI: 1.29-14.93).^25^ A cross-sectional study of 600 primary and nursery children in India found higher mean df-t (number of decayed and filled teeth) to be associated with poorer school performance when comparing groups with excellent and below average marks and average and below average marks.^26^

Janus and colleagues investigated the impact of poor oral health on school readiness in Canada (n = 576,264).^27^ Teachers completed the 103-item Early Development Instrument (EDI), a measure of children's ability to meet age-appropriate developmental expectations. It includes five general domains of development: physical health and wellbeing; social competence; emotional maturity, language and cognitive development; communication skills; and general knowledge. After adjusting for age, sex, special educational needs, English or French as first language, and neighbourhood socioeconomic status, children with teacher-reported unaddressed dental needs (UDNs) were more likely to be vulnerable on at least one EDI developmental domain compared to children without UDNs (OR: 8.434, 95% CI: 7.601-9.358; p <0.001).

An Australian cross-sectional study assessed the relationship between childhood health conditions, health service utilisation and subsequent academic performance in four- and five-year-old children (n = 24,678).^28^ It matched data from the 2008 School Entrant Health Questionnaire (completed by parents/carers) with the 2011 National Assessment Program - Literacy and Numeracy (NAPLAN) and controlled for confounders such as age, sex, language spoken at home, attendance at preschool and socioeconomic status in the analysis. Children with dental problems (not defined) were more likely to have a score at or below the national minimum in numeracy (OR: 1.2; 95% CI: 0.9-1.4) and more likely to have a score below the national minimum standard for reading (OR: 1.1; 95% CI: 0.9-1.3). Despite the lack of statistical significance in a large sample, the authors suggested that dental health, along with other health conditions, increased the risk of poor school performance.

Muirhead and Marcenes (2004) correlated various data in an ecological study in Wandsworth, London (n = 1,968).^29^ They analysed caries experience (five-year-old dmft), deprivation of school location (Jarman score), school performance (results in English, mathematics, and Linguistic Awareness of Reading Readiness Test [LARR]) and free school meals eligibility. Multiple linear regression analysis demonstrated associations between caries experience, deprivation of school location, school performance (English, mathematics and LARR) and proportion receiving free school meals. The authors reported LARR scores and the proportion of children receiving free school meals predicted mean school caries experience. As an ecological study the direction of this relationship cannot be established; however, the findings are relevant to the aim of this review.

## Discussion

The purpose of this rapid review was to determine if poor oral health of young children affects speech and language development, OHRQoL and school attendance and performance. Only one systematic review addressed the primary outcome of the impact of poor oral health on speech and language development. The review concluded that children with loss of primary anterior teeth were at higher risk of speech distortion than those without tooth loss.^15^ However, the two studies on which this was based had very small sample sizes and were reported as having high risk of bias. There is a need for new longitudinal, controlled observational studies of 0-5-year-old children with methodological rigour to determine if there is an association between premature loss of primary anterior teeth and speech and language development. Observational studies are also required to determine any association between untreated ECC and children undergoing extraction of multiple primary teeth and subsequent speech and language development issues. Opportunities should also be explored for the routine collection and evaluation of standardised developmental outcomes (including speech and language), alongside oral health outcomes.

While Liang and colleagues (2022) concluded that ECC is causally linked to psychomotor deficiency, they did acknowledge the role of confounders (age, diet and socioeconomic status) which they had not accounted for. Yet, they suggested that further studies are needed to disentangle this web of causation and to establish whether ECC exacerbates existing inequalities in speech and language development in children from lower socioeconomic backgrounds.^19^ Early years provision in the UK involves children aged 0-5 years, thus this was considered the population of interest for this review.^7^ However, in other countries, age at school entry differs; Liang and colleagues had study populations which included a small number of six-year-old children.^19^^,^^20^ As the proportions of this age group in their study populations were small (9% and <1%, respectively), it was decided to include these studies as the findings are still likely to be relevant to the UK and elsewhere.

ECC was found to be associated with reduced OHRQoL in preschool children, with a further reduction in OHRQoL seen in children as the severity of caries increased.^21^^,^^22^ Similar findings have been reported in two systematic reviews including children of wider age ranges and adolescents. Dental pain was found to have a negative impact on the OHRQoL of children and adolescents aged 0-19 years.^30^ Children aged 18 years and under with one or more decayed teeth had higher probability of poor school performance and attendance than caries-free children.^23^ In both reviews, the certainty of the evidence was low due to methodological limitations of the original study designs and the likelihood of confounding.^23^^,^^30^

Disease prevention, treatment and access to early learning opportunities are all essential for a young child to reach their developmental potential.^31^ Notwithstanding the limitations of existing evidence, findings of this review suggest that poor oral health is negatively associated with child OHRQoL, school attendance and school performance. Consequently, urgent action is needed to improve the oral health of the most vulnerable young children and to ensure early intervention so children are not prevented from reaching their full developmental potential. Collaboration will be needed between those organisations involved in promoting health, including oral health, and education of young children. Examples of such collaborations include inclusion of oral health in the work of family hubs and optimising implementation of supervised toothbrushing programmes in nurseries and schools.^32^^,^^33^^,^^34^ Further work will be needed to evaluate the impact of these initiatives.

The limitations of this rapid review must be considered. Searches were restricted to one database, eligible studies had to be written in English and published from 2000 onwards, and thus some studies relevant to the aim of the review may have been missed. Furthermore, a study protocol was not published and a quality assessment of the included studies was not conducted. The majority of the included studies addressed the secondary outcomes, highlighting substantial gaps in the literature of the impact of ECC and speech and language development. In addition, the majority of the included studies were primary observational studies rather than synthesised evidence, so risk of bias is likely to be high and many did not adequately control for confounders, including socioeconomic status, in their analyses.

## Conclusion

There is some evidence that poor oral health in young children is associated with negative impacts on development of speech and language, OHRQoL, school attendance and school performance. High-quality, observational, longitudinal research is required to establish the impact of poor oral health on the lives of young children. Strategies to improve oral health and enable early intervention with vulnerable children in this age group should be considered to ensure they meet their developmental potential.

### Supplementary Information

Supplementary Information (PDF 11KB)

## Data Availability

The data is available from the authors upon request.
