# Supplementary material for: Does poor oral health impact on young children's development? A rapid review
Source: Br Dent J. 2024 Aug 23;237(4):255–60. doi: 10.1038/s41415-024-7738-4 (PMC11343692; doi:10.1038/s41415-024-7738-4)
Supplement: Supplementary file 1 — Supplementary Information (PDF 11KB) [file 41415_2024_7738_MOESM1_ESM.pdf]

Supplementary material

Search strategy used for rapid review of evidence of association between poor oral health and development of young children (0-5 years)

1 Infant/

2 Child, Preschool/

3 (child* or infant or boy or girl or baby or toddler or newborn or babies or youngster or offspring or infancy or 0-5 years or early years or pre?school* or pre-school children or young child* or primary or reception or kindergarten or p?ediatric).tw.

4 1 or 2 or 3

5 Oral Health/

6 Tooth Loss/

7 Dental Caries/

8 (oral health or dent* or tooth or teeth* or caries or carious or "dmft" or nursing caries or bottle caries or early childhood caries or dental care or poor oral health or early tooth loss or premature tooth loss).tw.

9 5 or 6 or 7 or 8

10 4 and 9

11 Speech/

12 Child Language/

13 Language Development/

14 Child Development/

15 Educational Measurement/

16 Absenteeism/ and Schools/

17 Quality of life/

18 (speech or language or literacy or communicat* or linguistic or diction or phonic or numera* or vocabulary or articulat* or enunciat* or word?gap or talk* or speak* or spoken or cognit* or intelligen* or IQ).tw.

19 (((school* or education*) adj (attend* or absent* or absence or readiness or progress* or attain* or achiev* or ability)) or school attendance or school performance).tw.

20 11 or 12 or 13 or 14 or 15 or 16 or 17 or 18 or 19

21 10 and 20

22 limit 21 to (english language and humans and yr="2000 -Current")
